# Vitamin A5: Evidence, Definitions, Gaps, and Future Directions

**DOI:** 10.3390/nu17142317

**Published:** 2025-07-14

**Authors:** Torsten Bohn, Sascha Rohn, Volker Böhm, Marta Despotovic, Angel R. de Lera, Wojciech Krezel, Omer Kucuk, Diána Bánáti, Ralph Rühl

**Affiliations:** 1 Nutrition and Health Research Group, Department of Precision Health, Luxembourg Institute of Health, 1 AB, Rue Thomas Edison, 1445 Strassen, Luxembourg; torsten.bohn@lih.lu; 2 Department of Food Chemistry and Analytics, Institute of Food Technology and Food Chemistry, Technische Universität Berlin, 13355 Berlin, Germany; rohn@tu-berlin.de; 3 Institute of Nutritional Sciences, Friedrich Schiller University Jena, 07743 Jena, Germany; volker.boehm@uni-jena.de; 4 Department of Nutrition and Metabolism, Institute for Medical Research, 11000 Belgrade, Serbia; martadespotovic@yahoo.com; 5 Departamento de Química Orgánica, Facultade de Química, CINBIO and IBIV, Universidade de Vigo, Campus As Lagoas-Marcosende, 36310 Vigo, Spain; qolera@uvigo.es; 6 Institut de Génétique et de Biologie Moléculaire et Cellulaire (IGBMC), Inserm U1258, CNRS UMR 7104, Université de Strasbourg, 67400 Illkirch, France; krezel@igbmc.fr; 7 Department of Hematology and Medical Oncology, Winship Cancer Institute, Emory University School of Medicine, Atlanta, GA 30322, USA; omerkucukmd@gmail.com; 8 Department of Food Engineering, Faculty of Engineering, University of Szeged, H-6725 Szeged, Hungary; diana.banati@gmail.com; 9 CISCAREX UG, 13351 Berlin, Germany

**Keywords:** mental health, neurodegenerative diseases, healthy diet, leafy vegetables, longevity

## Abstract

With the emergence of a new vitamin concept—vitamin A5—it is essential to first clarify the basic definition of vitamins, particularly vitamin A. This article summarizes the foundational concepts and definitions of vitamins with particular relevance to the discovery, establishment, and categorization of new vitamin concepts. Vitamin A5 was discovered 80 years after the last vitamin was identified. It serves as an umbrella term for the dietary precursors 9-*cis*-β,β-carotene and 9-*cis*-13,14-dihydroretinol for the endogenous activator of the nuclear hormone receptor RXR, 9-*cis*-13,14-dihydroretinoic acid. However, several questions arise: Which criteria are typically used to identify a substance as a vitamin? How does vitamin A5 fit into the sometimes misleading definition of vitamin A? This review summarizes key findings and provides a comprehensive assessment of the current understanding, concluding that (a) vitamin A5 is a newly identified micronutrient that plays an important role in the prevention of diet-related diseases and (b) vitamin A5 is an important micronutrient that provides a plausible, mechanistic explanation for why a Western lifestyle diet low in vegetables and especially leafy vegetables can lead to a high prevalence of Western-lifestyle diseases, particularly neurological diseases and poor mental health.

## 1. Introduction—How Do You “Discover” and Establish a Vitamin? A Brief Historical Review

Vitamins are typically associated with specific deficiency symptoms. However, historically, the precise onset of vitamin deficiency symptoms and the identification of the factors responsible have not always been clear or straightforward. It often begins with a theory, suggesting that physiological dysfunction or even existing pathological indications can be reversibly prevented by food components. Experimental validations start with preliminary animal studies, which establish potential vitamin deficiencies through non-optimal, reproducible, and standardized experimental methods. Subsequently, these deficient animals are supplemented with specific food extracts, or isolated compounds or selected substances, that are believed to be responsible for correcting the deficiency.

A century ago, experiments on humans were typically conducted quickly and with relative ease. On a “let’s get started” basis, a number of “volunteers” and / or patients were simply “recruited” and treated with laboratory extracts [1]. Targeted clinical interventions in humans—such as (a) inducing specific vitamin deficiencies, (b) investigating the correlation between the intake or concentration of a specific vitamin in the body and markers of dysfunction, or (c) supplementing vitamin-deficient “volunteers” or patients—were not carried out in detail until much later. The delay was due to the lack of analytical methods to identify specific deficiencies through laboratory techniques and to determine the effects of substitutions analytically and mechanistically, as exemplified for vitamin C in Maxfield and Maxfield [2].

At that time, many aspects of vitamin discovery were supposedly much simpler: there were no restrictions or guidelines for animal experiments, no ethical considerations for clinical trials, no clinics with high overhead costs, and no restrictive government agencies. However, similar problems existed then as they do now, such as a lack of funding to conduct trials. Clinical interventions involving targeted nutritional deficiencies and supplementation experiments were often carried out “en passant” on patients, prisoners of war, or prison inmates [3,4]. These experiments, which now seem bizarre, were later uncritically incorporated into national and international vitamin and food references [5,6]. The problem is that the existing national and international vitamin references contain numerous inconsistencies, which may be regarded as inviolable from a historical perspective but not necessarily from a scientific one.

Only much later, after the discovery of additional vitamins and the establishment of vitamin concepts, starting from the 1960s [6], uniform criteria and definitions for vitamins and micronutrients were established by the World Health Organization (WHO) / Food and Agricultural Organization (FAO) Expert Groups [6], and reference intakes were proposed [7,8,9]. Furthermore, after the establishment of the European Food Safety Authority (EFSA) in 2002, regulations on novel foods [10] and on substance-specific health claims were also developed [11].

The purpose of this review article is to conclusively summarize the definition of terms such as vitamin A and vitamins in general, to further summarize and evaluate what data is present or missing to conclusively categorize vitamin A5 as a vitamin.

## 2. What Is a Vitamin as Defined by the WHO and EFSA?

**Figure 1:**
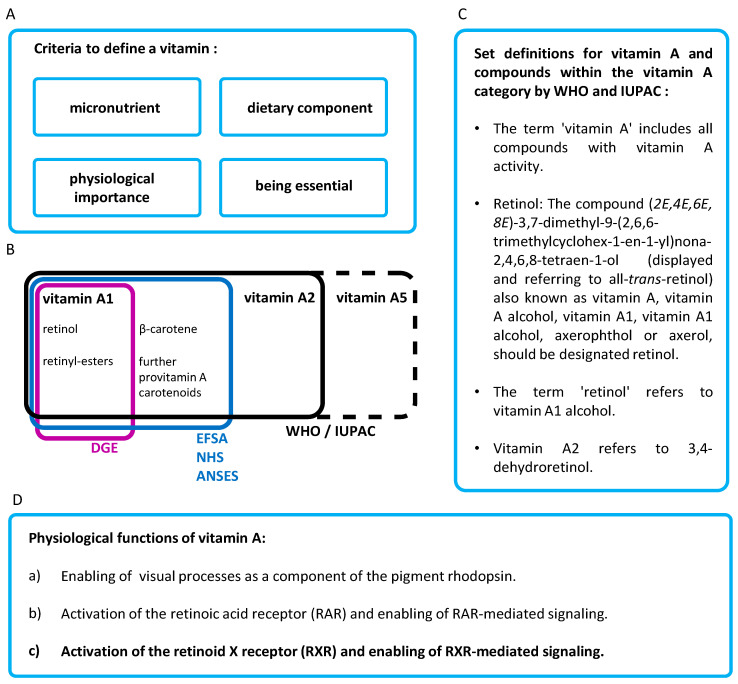
(**A**) Summary of the criteria that define a vitamin [6], (**B**) the definition of vitamin A by various organizations. World Health Organization (WHO), International Union of Pure and Applied Chemistry (IUPAC), European Food Safety Authority (EFSA), German Nutrition Society (*Deutsche Gesellschaft für Ernährung*, DGE), British National Health Service (NHS) and the French Agency for Food, Environmental and Occupational Health & Safety (*Agence nationale de sécurité sanitaire de l’alimentation*, ANSES), (**C**) summary of definitions for vitamin A and compounds within the vitamin A category and (**D**) summarized criteria of vitamin A-mediated activity.

The WHO states: “*Definition of terms: The following definitions relate to the micronutrient intake from food and water required to promote optimal health, that is, prevent vitamin and mineral deficiency and avoid the consequences of excess*” [6] (Figure 1A).

EFSA’s definition of a vitamin is as follows: “*Dietary substance needed in very small amounts to support normal growth and maintenance of health in humans and animals. Most vitamins are “essential” as they are not made within the body*” [12]. The definition is somewhat mitigated by the addition of the word “most” before the word “vitamins”, presumably to avoid the withdrawal of the status of a vitamin from the non-essential “vitamin” D [13], based on an overly strict vitamin definition.

In total, four criteria are used to define a vitamin. A vitamin must be (a) a micronutrient, (b) a component of the diet, (c) transmit a physiologically important function, and (d) be essential (according to the EFSA, “*as they are not made in the body*”) (Figure 1A).

## 3. What Is the Definition of Vitamin A?

### 3.1. Vitamin A Definitions of the EFSA, the British National Health Service (NHS) and the French Agency for Food, Environmental and Occupational Health & Safety (Agence Nationale de Sécurité Sanitaire de L’alimentation, ANSES)

*“Vitamin A is a fat-soluble vitamin obtained from the diet either as preformed vitamin A (mainly retinol and retinyl esters) in foods of animal origin, or as provitamin A carotenoids in plant-derived foods. Foods rich in vitamin A include meat, butter, retinol-enriched margarine, dairy products, eggs, and vegetables and fruits such as sweet potatoes, carrots, pumpkins, dark green leafy vegetables, sweet red peppers, mangoes and melons.”*

[12]

The term “vitamin A” therefore includes the substances retinol, various retinyl esters, and various provitamin A carotenoids (Figure 1B) [14,15]. This definition is also used by the NHS in the United Kingdom [16] and the ANSES in France [17].

### 3.2. Vitamin A Definition by the German Nutrition Society (Deutsche Gesellschaft für Ernährung, DGE)

The DGE defines the term vitamin A as follows: “*Vitamin A is a vital (essential), fat-soluble nutrient that is necessary for many biological processes such as vision, immune function, cell differentiation and embryonic development. The term vitamin A describes a group of compounds that have a vitamin A effect. The main active form is retinol”* and *“**Plants contain a number of provitamin A carotenoids that can be converted to vitamin A to varying degrees.** The most important provitamin A for human vitamin A intake is β-carotene, as it has a high rate of conversion to retinol and is the most abundant.* ***Provitamin A carotenoids are not essential****, but are particularly important for maintaining an adequate vitamin A status, especially in predominantly vegetarian or vegan diets*.”

The term “vitamin A” as defined by the DGE includes only retinol and retinyl esters, and it does not include provitamin A carotenoids. According to this definition, a paradox is created based on the following contradictory statements: (a) “*vitamin A describes a group of compounds that have vitamin A effects*” and (b) “*provitamin A carotenoids can be converted to vitamin A*”. However, as provitamin A carotenoids mediate vitamin A effects directly, i.e., without direct conversion to retinol, and thus provide vitamin A activity, based on dozens of scientific studies [15,18,19,20], provitamin A carotenoids belong to the vitamin A family, as also defined by the WHO and EFSA. Therefore, the conversion of “vitamin A” (in the form of provitamin A) to “vitamin A” is a paradox. It is suspected that the terms “retinol”, “vitamin A”, and “vitamin A1” have been equated for simplification by the DGE, although they describe different things. A further statement by the DGE that provitamin A carotenoids are not essential, although they belong to the vitamin A family, is therefore another paradox in the terminology used.

### 3.3. Vitamin A Definition of the WHO and the International Union of Pure and Applied Chemistry (IUPAC)

To avoid misinterpretations for already existing simplifications in the current [21] and initial [22] IUPAC definition, the problem of an unclear simplification was already addressed and a clear recommendation was given: “*Retinol: The compound (2E,4E,6E,8E)-3,7-dimethyl-9-(2,6,6-trimethylcyclohex-l-en-1-yl)nona-2,4,6,8-tetraen-1-ol (displayed and referring to all-trans-retinol) also known as vitamin A, vitamin A alcohol, vitamin A1, vitamin A1 alcohol, axerophthol or axerol, should be designated retinol*.” (Figure 1C).

According to the IUPAC definition [21,22], the vitamin A family includes the substance groups vitamin A1 and vitamin A2. In the first WHO / FAO communications in 1967 [5], a definition was set up, clearly stating the following: “*The term ‘vitamin A’ includes all compounds with vitamin A activity.*” and “*The term ‘retinol’ refers to vitamin A1 alcohol*.” (Figure 1C). This provided a clear definition of vitamin A and vitamin A1 and clearly distinguished these two terms from each other. This WHO / FAO definition [5] referred to and confirmed the initial IUPAC definition [22]. A definition for vitamin A2 was also provided (“*Vitamin A2 refers to 3,4-dehydroretinol*.” [5,22]) and is further summarized in [23].

These definitions are still valid and were not further officially changed by subsequent WHO / FAO communications. However, in the WHO communications from 1988 [24] onwards, the term “vitamin A” was not newly defined but was equated with the terms “retinol” and “vitamin A1”. From a legal and general standpoint, the 1967 WHO definition has never been revoked; rather, it has been “optimized” and simplified against the official logical recommendations by IUPAC and the WHO / FAO. This simplification, while misleading and imprecise, of the vitamin A definition was then further even adopted by other national and international organizations.

According to the official IUPAC and WHO / FAO definitions, in addition to retinol, retinyl esters, and provitamin A, there are subgroups of vitamin A, namely vitamin A1 and A2, with the potential for other relevant subgroups to be recognized in the future. These subgroups (vitamin A1 and A2) have not been distinguished or included in further dietary guidelines, such as those based on current recommendations from the EFSA, alternative national organizations, or even the WHO. Only the officially IUPAC definition [21] clearly defines the terms correctly and does not adhere to any unclear misleading simplifications.

### 3.4. Vitamin A2—Forgotten or Even Suppressed on Purpose?

Vitamin A2 (3,4-dehydroretinol [23], first identified in fish oils, was discovered in the late 1920s [25,26]. It can functionally replace vitamin A1 in preventing general vitamin A deficiency [27]. Despite its potential, vitamin A2 has largely faded from scientific focus: over the past 20 years, only 30 scientific publications listed in the PubMed database (status April 2025) mention the term “vitamin A2,” compared to more than 60,000 for general “vitamin A.” As a result, it appears to have become largely overlooked—or even “suppressed” on purpose—to fit into the currently used simplified definition of vitamin A. This exclusion is likely due to the simplifications and the relatively minor role vitamin A2 likely plays in the human food chain.

The term “vitamin A1” has often been, and continues to be, partially equated with the term “vitamin A”—a practice that is scientifically and regulatory inaccurate. However, this conflation has not been widely questioned or considered problematic [23,28,29,30,31,32].

### 3.5. The Vitamin A Status of Vitamin A(1) Aldehyde and Vitamin A(1) Acid

The substance retinal is also defined by the EFSA as vitamin A [33] because retinal can be metabolized to retinol (all-*trans*-) and is therefore also included under the definition of vitamin A. It also appears to be recognized that retinal, primarily found in eyes, is a relevant dietary component.

Retinoic acid (all*-trans*-), also known as vitamin A / A(1) acid, is another compound to be considered. It is not present in significant concentrations in commonly consumed foods and cannot be metabolized to retinol (all-*trans*-) in the human body. A key function of vitamin A, which is mediated by retinal in the visual perception, can therefore not be mediated. Thus, two important criteria for classifying a substance as vitamin A are not fulfilled in the case of retinoic acid.

### 3.6. Summary of the Definition of Vitamins in General and Vitamin A in Particular

The issue is that different national and international professional bodies do not consistently define according to the correct and official definition of “vitamin A”. Moreover, it is used in a paradoxical manner. Three main problems occurring with the current situation regarding differing definitions are summarized below:

(i) Based on the information provided by the EFSA and WHO / FAO, as well as the current state of scientific knowledge (reflected in numerous publications and reviews [14,15,20]), provitamin A is considered part of the vitamin A family, and vitamin A is essential. This understanding is grounded in the general definition of vitamins provided by the WHO / FAO and EFSA [6,12,24] and supported by multiple scientific studies and reports [15,18,19,20].

(ii) Another issue arises from the simplification and using the terms “retinol”, “vitamin A,” and “vitamin A1”. These current inconsistencies in the definition of vitamin A lead to significant interpretational challenges and prevent a proper differentiation between subgroups, such as vitamin A1, vitamin A2, and other potential additional subclasses, limiting a more nuanced understanding of the vitamin A group. However, this simplification practice, foreseen in the early and current IUPAC definitions [21,22] as well as the valid WHO / FAO definitions [5], was recently summarized in detail [23].

(iii) A further complication is the continued use of vitamin A alcohol / retinol as the reference compound for vitamin A-mediated activity and subsequent dietary recommendations. This is problematic because the biologically active form, all-*trans*-retinoic acid (ATRA), and its role in retinoic acid receptor (RAR)-mediated signaling are more relevant to vitamin A function.

## 4. The biological / physiological Mechanisms of Action of Vitamin A

**Figure 2:**
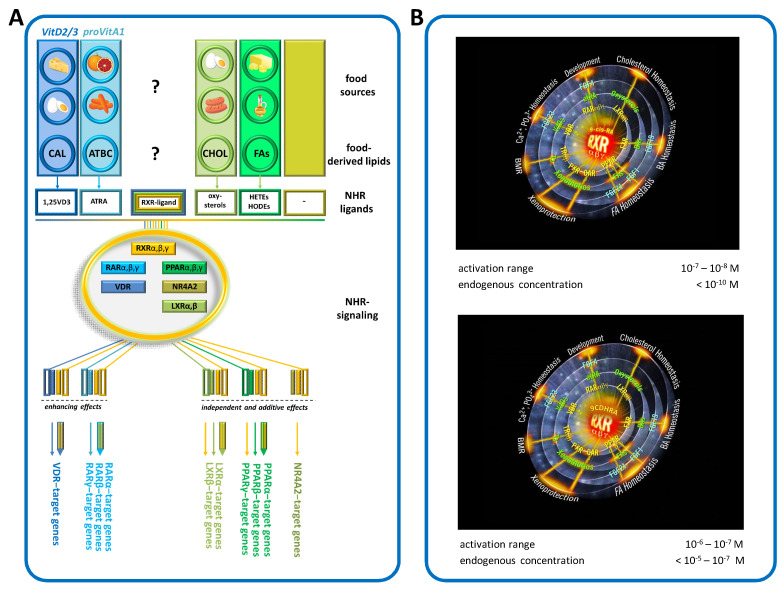
(**A**) Summary of RXR-controlled signaling, modified from earlier [23]. Abbreviations: Nuclear hormone receptor (NHR), peroxisome proliferator-activated receptors (PPARs), vitamin D receptor (VDR), liver X receptor (LXR), “nuclear receptor subfamily 4 Group A Member 2” (NR4A2), retinoid X receptor (RXR), provitamin A1 (proVitA1), vitamin (Vit), all-*trans*-retinoic acid (ATRA) and 1,25 dihydroxy-vitamin D3 (1,25VD3). (**B**) Top figure, 9CRA as “BIG BANG” and bottom figure, 9CDHRA as “BIG BANG” for the physiologically important RXR signaling mediated by interaction with a variety of nuclear hormone receptors and their important physiological functions in the human organism [31,35].

The biological mechanisms of action for optimal health and the prevention of vitamin A deficiency include three biological / physiological vital / essential mechanisms (Figure 1D):

(i) The action of retinal (11-*cis*-retinal) as a component of the pigment rhodopsin in the visual process.

(ii) Activation of the retinoic acid receptor (RAR) and subsequent RAR-mediated signaling initiated by all-*trans*-retinoic acid. This RAR-mediated signaling involves important physiological functions of vitamin A in the immune system, embryonic development, cellular differentiation and proliferation, and other biochemical processes.

(iii) Activation of the retinoid X receptor (RXR) and subsequent RXR-mediated signaling. The RXR is the respective partner for the RAR; as such, it is also an important determinant for RAR-mediated signaling. It is also a ligand-dependent partner in signaling vitamin D via the vitamin D receptor (VDR); of thyroid hormones via the thyroid hormone receptor (TR); of cholesterol derivatives via the liver X receptors (LXRs); of fatty acids and lipid mediators via the peroxisome proliferator-activated receptors (PPARs); and in other signaling instances, such as via alternative nuclear hormone receptors, e.g., the “nuclear receptor subfamily 4 group A member 2” (NR4A2 / NURR1) (Figure 2A).

The RXR is the most important physiological master switch, enabling a multitude of signaling pathways via numerous nuclear hormone receptors [34]. An endogenous RXR ligand is therefore the most important dietary-derived substance that enables and controls all these signaling cascades [35,36].

## 5. The Missing Piece of the Vitamin A Puzzle: The Food-Derived Precursors for the Ligand-Activation of Vitamin A—Controlled RXR-Mediated Signaling

Based on experiments in animal models, the most important part of a vitamin A-mediated effect is vitamin A—RXR-mediated signaling [34,37]. This vitamin A—RXR-mediated signaling effect includes not only the vitamin A—RXR—RAR-mediated effect, which is important for general and embryonic development, but also the control of physiological processes such as cellular differentiation, apoptosis and proliferation with essential importance for the immune system, and the maintenance of skin, bones, and other organs.

The importance of vitamin A—RXR-mediated signaling extends much further, to the general control of processes mediated by other nuclear hormone receptors, such as VDR, TR, LXRs, PPARs, and NR4A2, which are responsible for, among other things, the general maintenance and control of our body’s metabolism [35]. Some of these mechanisms are vital to mammalian organisms, while others are not immediately essential. Using the example of the control of insulin secretion mediated by vitamin A—RXR signaling [34,38], the long-term dysregulation of vitamin A—RXR signaling can have serious consequences that only occur after a long period of dysregulation, as in the case of diabetes mellitus [39]. This disease is a chronic, treatable disease that severely limits the quality of life but is not fatal and immediately leading to death. In this case, vitamin A—RXR signaling would not be proximately essential. These consequences therefore only occur after a (very) long period of dysregulation, i.e., not immediately, comparable to other known chronic diseases related to Western lifestyle, such as cardiovascular disease, atherosclerosis, suboptimal mental health, and an increased prevalence of a variety of neurological diseases, with a focus on neurodegenerative diseases [40,41].

These disorders are different from those caused by a deficiency of B-vitamins. These water-soluble B vitamins are not stored in organisms in significant quantities (except for vitamin B12) and must therefore be rather taken in on a daily basis. In the case of vitamin A and vitamin A—RXR signaling, an acute effect is difficult to determine and difficult to correlate with a current daily vitamin intake. In the context of vitamin A and vitamin A deficiency, long-term effects relevant to chronic diseases must also be considered, even when these chronic diseases are not immediately fatal and can be explained by an essential vitamin effect.

The important question is: how does vitamin A mediate this RXR activation and subsequent RXR-mediated signaling?

## 6. The Controversial “Phantom Ligand” for RXR, 9-*cis*-Retinoic Acid, and Other Potential Alternative Endogenous RXR-Ligands

9-*cis*-Retinoic acid (9CRA) was independently “identified” by two working groups and described as the endogenous ligand for the activation of the RXRs [42,43]. These studies were carried out in the early 1990s, using the most up-to-date analytical techniques available at the time. However, these techniques are now considered to be rather unselective and insensitive analytical methods based on the current state of the art. The detection of endogenous 9CRA in humans could not be carried out or at least not clearly, as recently summarized [18]. Only decades later, a working group was able to identify “9CRA” in humans, using liquid chromatography coupled to mass spectrometry (LC-MS), but again, this was not unambiguously and was only at concentrations far below the level required for RXR activation [44]. Another problem was that many alternative substances co-elute with 9CRA in the analytical chromatographic methods used [29,32,45,46], and the clear identification and quantification of the analytical peak as 9CRA was not yet possible [45].

To unambiguously detect an endogenous substance, ideally two or more independent analytical detection methods need to be employed. In the case of 9CRA, separation by HPLC should be followed by different detection techniques such as a selective MS / MS methodology and a diode array detector [47]. Based on this need for the parallel detection of 9CRA by two independent methods, many publications that have detected and quantified 9CRA earlier must be questioned [43,48]. The interpretation that 9CRA has not been unambiguously identified and that it is not the endogenously occurring RXR ligand is currently accepted by experts in the field of retinoid analysis, as reviewed previously [18,29,30,32].

There are also other potential endogenous RXR ligands in discussion with non-conclusive and unlikely physiological / nutritional impact, summarized in [30]. These are the “free” non-esterified fatty acids such as phytanic acid, palmitoleic acid, linoleic acids, docosapentaenoic acid, oleic acid, and docosahexaenoic acid (DHA) [49,50,51].

Focusing on the most likely candidate, DHA, its endogenous / physiological nutritional relevant levels in the mammalian and especially the human organism are not known, because of an unclear differentiation between a targeted determination of DHA being present in high levels as DHA-ester as well as the free non-esterified DHA [50], especially in target organs, cells, and the cell nucleus. Only the free non-esterified DHA might interact endogenously on the nuclear levels with the nuclear hormone receptor RXR, as was preliminary experimentally determined in artificial transfected reporter cell line models to enable transcriptional activation by high levels of DHA, which were reported to be in the range of 10 µM [52], 66 µM [49], or even >100 µM [50]. In mammalian models, as a more complex non-nutritional like scenario, *intra*-peritoneal DHA administration resulted in the initiation of specific RXR-mediated signaling [53]. A clear causal connection between added DHA, as it is, and further DHA—RXR-mediated signaling was not examined in mice and especially also humans. Alternative mechanisms via the DHA metabolites, as protectins and maresins [30,54,55], as well as the secondary DHA-induced pathway via activating alternative nuclear hormone receptor pathways [54,55,56] (thereby influencing RXR-mediated signaling), are a more likely scenario [57,58]; these are partly under current investigation. Unfortunately, besides experimental data from reporter cell constructs and still-fragile analytical data for free DHA in mammals, there are multiple interpretations of how this link between DHA and RXR signaling might be transmitted. These alternatives should be investigated in more detail instead of being fixed on the unlikely scenario of the simplified option: DHA being present in food, further ingested into our organism, and directly interacting with the RXR on a nuclear level.

While focusing on the most likely alternative candidate, 9CRA, the questions of whether (a) 9CRA is present in the human organism and whether (b) there is a controlled enzymatic synthesis in the human organism (again based on inadequate analysis) have not yet been clearly answered; they are speculative and therefore not a proven scientific fact. Unfortunately, this fragile “9CRA—endogenous RXR ligand” hypothesis is still being promoted by many nutritional experts that have limited experience in the field of retinoid analysis and are not directly involved in this complex subject.

In 1992, the Ron Evans group and colleagues identified 9CRA as the endogenous RXR ligand [43] and later described 9CRA as the “BIG BANG” in nuclear hormone receptor research [59]. In a review article in 2022, Evans revised his statement based on the work of Krezel et al. [60]*:* “*A search for a higher affinity retinoid for RXR led to the identification of the 9-cis isomer of RA (9-cis-RA) as a suitable ligand* [42,43]. *Given its low abundance in tissues and high affinity of the RARs, the exact role that endogenous 9-cis-RA plays as an RXR ligand in retinoid signaling in vivo has remained elusive. However, it has recently been reported that a retinoid related to 9-cis-RA present at high endogenous levels in mice, namely 9-cis-13,14-dihydroretinoic acid, binds and transactivates all three RXRs at physiological concentrations* [46]” (Figure 2B).

In sum, the status of 9CRA as the physiological ligand for the RXR with an enzymatically regulated metabolic pathway is still very present in the scientific literature, but this hypothesis remains very fragile and has been already revised by Dr. Ron Evans [59], based on the later work of Krezel, Rühl, and de Lera [60] around the discovery of the vitamin A5 pathway [32,46]. Other candidates as physiologically and nutritionally relevant endogenous RXR activators such as DHA are also unlikely due to the lack of knowledge of an overlapping activity range vs. physiological / nutritional occurring levels. There is also a complete lack of understanding of the mammalian and—most importantly—human relevance of the relationship between DHA in food and endogenous RXR-mediated signaling [29,30,32].

## 7. Vitamin A5 as a New Conclusive Candidate Being a Nutritional Precursor for an Endogenous-Relevant RXR Ligand

The evidence for the endogenous occurrence of 9-*cis*-13,14-dihydroretinoic acid (9CDHRA) and its potential activation of the nuclear hormone receptor (NHR) and the RXRs, as well as its function as a new lipid hormone, formed the basis for the postulation of the vitamin A5 concept in 2018 [32]. This postulate was also made considering the unclear and fragile status of the “9CRA—endogenous RXR ligand” hypothesis and an embedding in the current vitamin A concept [31]. These aspects were also embedded as scientific consensus in the current vitamin A concept in review papers of the EU COST action “EUROCAROTEN”, through a review article with 21 internationally experienced carotenoid experts as co-authors [14] and another position paper of the EU COST action “POSITIVE” [20].

Work on the identification and action of 9CDHRA has identified a paramount new component that can, for the first time, conclusively explain an important physiological mechanism of action of vitamin A: the RXR signaling pathway. A “food to ligand to vitamin A–RXR-mediated effect” by 9CDHRA and the postulated vitamin A5 concept comprised the first complete representation that logically explained food-related RXR activation and employed it as a “food to action” concept.

The basis for this was the identification of 9CDHRA as an active RXR ligand and hormone-like substance, which also occurs in physiologically and nutritionally relevant concentrations in the mammalian model [46] and also in humans [61]. These studies with 9CDHRA also included evidence for data related to the binding of 9CDHRA to RXR and a resulting RXR-mediated effect.

## 8. The Vitamin A5 Concept: From the Postulate of the Vitamin to the Confirmation of the Concept

In order to ensure a scientifically transparent view concerning vitamin A in general and vitamin A5 specifically that is relevant for the entire population, independent national and international experts in the field of vitamin A / carotenoids summarized the current data in this review on vitamin A / A5 in the following chapters, in order to enable a broader and balanced presentation of the legal and scientific basis.

**To confirm the food-induced RXR-mediated mechanism, the following experiments were performed:**

(a) **Genetic animal models like the specific RXRγ- and RBP1 / CRBP1-“knock out” mice** were generated to investigate the role of RXR signaling, mainly focusing on animal development [62], and used in further experimental procedures, where the cognitive functions of animals were examined in delayed tasks (maze-based task) similar to those employed in human clinical studies [46,53,60]. These experimental procedures were used to selectively detect RXR-mediated signaling effects [46,60]. Therefore, specific experimental mice models for “knocking out” RXRs—in the present case, the RXRγ-receptor—were created. In these animals, the specific coding gene for RXRγ was made inoperative [62]. This experimental procedure is commonly simplified with the term gene-“knock out” model. These RXRγ-“knock out” animals displayed cognitive deficits [46,53,60], indicating that RXR-mediated signaling was important for cognition and further suggesting that an endogenous RXR ligand likely might be important for enabling this RXR-mediated signaling. This hypothesis was further addressed using experimental models. It was noticed that RBP1 / CRBP1-“knock out” mice display similar cognitive dysfunction [46] to that found in the RXRγ-“knock out” animals. However, in contrast to RXRγ-“knock out” mice, which remain insensitive to synthetic RXR ligands, the memory and affective deficits of RBP1 / CRBP1-“knock out” mice were normalized using such treatments, pointing to the potential deficit of an endogenous ligand of RXRs in RBP1 / CRBP1-“knock out” mice. Using these animal models and observed short-term memory and affective behaviors as markers of RXRγ signaling, 9CDHRA was identified as the endogenous-relevant RXR-ligand [46]. The previously assumed optional ligand, 9CRA, could not be detected in the serum or brains of wild-type, RXRγ-, or RBP1 / CRBP1-“knock out” animals [46], though it can, in general, be easily detected, such as after ATRA treatments [53,63].

Food restriction-based methods to evaluate an impact of vitamin A5 deficiency on animal health are far from being easy to carry out. This is due to (a) a larger overlap in nutritional sources of vitamin A(1) and vitamin A5 [18,31,64], (b) fragile endogenous derivatives [32,43,65], (c) a partly overlapping RAR- and RXR-activation potential of various target endogenous retinoids [43,46], and (d) the highly crucial functions of RAR- and RXR-mediated pathways during embryonic development in mammals [66].

Further studies of RXR-subtype mouse “knock out” models where the RXRs can be specifically deleted in the brain via the Cre-LoxP technology [67] will be essential to better understand physiological functions controlled by RXRs in the central nervous system. Specific vitamin A1 or vitamin A5 deprivation and targeted re-supplementation will be compared in these specific “knock out” models to disentangle the transmission of RXR- or RAR-specific signaling in mammals.

Further experimental models specifically addressing the postnatal functionality of RXRs, while maintaining the functionality of the RXRs during the critical embryonic development phase, are based on tamoxifen-inducible RXR-“knock out” CreERT2-depending technologies [68] and will allow for investigating RXR functions in specific cells and organs, or in whole organisms [69]. These strategies were partly executed for alternative enzymes and receptors and specific organs with relevance for RAR–RXR-mediated signaling [70,71,72,73,74]. These laborious experiments might link the crucial need for RXRs involved in RXR signaling during embryonic development and the crucial control of systemic lipid and glucose homeostasis in adult mammals. Although the development of brain-specific RXR-“knock out” models is in progress using different strategies, currently no specific RXR single-subtype (RXRα, RXRβ, RXRγ) or even double- or triple-subtype-specific CreERT2-“knock out” mouse models [68,69] exist; they must be developed from scratch, including further specific dietary interventions for selective vitamin A-subtype deficiencies and further vitamin A-subtype-specific re-supplementations. These highly laborious experiments might partly lead to further insight into how individual vitamin A subclasses induce specific food-mediated RXR or RAR signaling.

(b) **The activation and initiation of RXR-mediated effects through the administration of vitamin A5 alcohol / 9-*cis*-13,14-dihydroretinol (9CDHROL) to mice** was also confirmed [60]. Notably, 9CDHROL is the dietary precursor of 9CDHRA, confirming the vitamin A5 concept. It was clearly shown that 9CDHROL is the direct dietary precursor for the endogenous ligand 9CDHRA, which initiates this selective vitamin A-specific RXR signaling.

(c) Further animal and cell culture experiments were carried out to identify other dietary precursors of 9CDHRA. It was found that the **substances 9-*cis*-13,14-dihydro-β,β-carotene (9CDHBC) and 9-*cis*-β,β-carotene (9CBC) are also proximate dietary precursors of 9CDHRA** and therefore have a vitamin A5 function and belong to the vitamin A5 family [60]. An extract rich in 9CBC, as provitamin A5, even transmits RXR-selective activities in supplemented animals, including improved cognition and decreased Alzheimer’s-disease neuropathology, such as reduced prevalence on β-amyloid plaques and tau hyperphosphorylation as well as neuro-inflammation [75].

(d) The substances **9CDHROL, 9CDHBC, and 9CBC** dealt with in these publications [46,60] **have also been detected in humans and in the human food chain**.

(e) It has also been shown that in mice, **vitamin A1 in the form of retinol (all-*trans-*) and provitamin A1 in the form of all-*trans*-β,β-carotene cannot be converted into vitamin A5 / provitamin A5** or the active ligand of vitamin A5 (9CDHRA). However, in a biological sense, it is correct to say that a very limited conversion takes place, which is not considered to be physiologically or nutritionally relevant. Furthermore, based on many scientific studies in the field of carotenoids / vitamin A, it can be assumed that this situation in mice is similar to that in humans and only corresponds to the conversion of one essential micronutrient, vitamin A1, into another essential micronutrient, vitamin A5.

**Figure 3:**
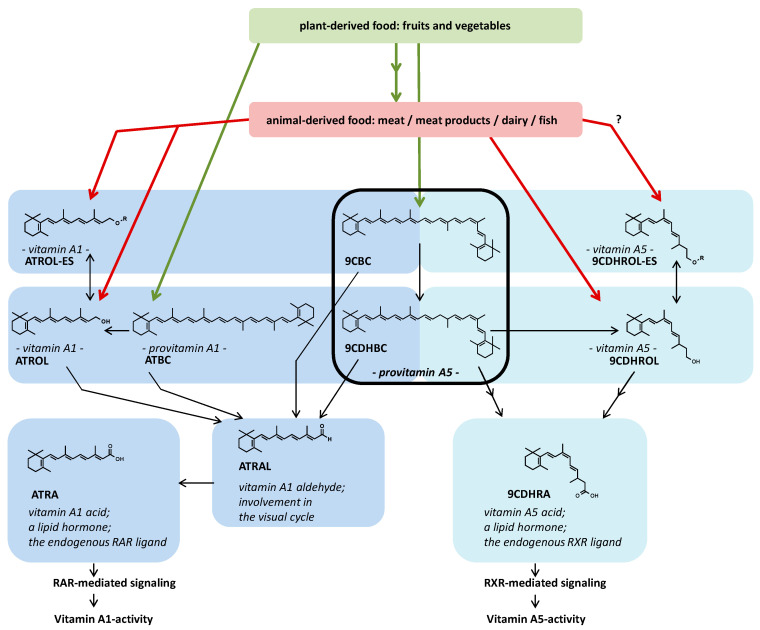
The new updated vitamin A concept with the embedding of vitamin A5 [60]. Abbreviations: Retinoid X receptor (RXR), retinoic acid receptor (RAR), all-*trans*-retinoic acid (ATRA), all-*trans*-retinol (ATROL), all-*trans*-β,β-carotene (ATBC), all-*trans*-retinyl ester (ATROL-ES), all-*trans*-retinal (ATRAL), 9-*cis*-β,β-carotene (9CBC), 9-*cis*-13,14-dihydro-β,β-carotene (9CDHBC), 9-*cis*-13,14-dihydroretinol (9CDHROL), 9-*cis*-13,14-dihydroretinyl ester (9CDHROL-ES), 9-*cis*-13,14-dihydroretinoic acid (9CDHRA).

Figure 3 portrays the metabolic concept of vitamin A5 from “food to vitamin to active ligand to selective vitamin A-specific RXR-mediated signaling” and illustrates the selectivity of precursor usage for the generation of ATRA and 9CDHRA (Figure 3, presented as Figure 5 in [60]).

(f) **The creation of a “food via mechanism to action” concept** to scientifically describe the protective effect of a healthy diet containing sufficient vitamin A5. In this case, vegetables, especially leafy vegetables, have been described as a protective factor against certain diseases of Westernized civilization, such as a variety of neurological diseases.

Currently, there is evidence that vitamin A5 is the nutritionally relevant substance to generate selective “vitamin A—RXR specific—RXR-mediated signaling. There is currently no alternative and proven “food to ligand to RXR signaling” concept other than this present vitamin A5 concept. Furthermore, the RXR-specific effect of vitamin A5 has been confirmed with relevance to the brain (present in Figure 3F of [60]). Further studies confirm important functions of vitamin A5 in cognition, anxiety, and depression, i.e., important functions directly mediated in the brain [76], as well as vitamin A5 acid for myelination in general and specifically for protection from de-myelination, the initiation of re-myelination [77], and protection from neuro-inflammation / glaucoma [78].

## 9. Embedding of Vitamin A5 in the Present Context of Vitamin A

“Vitamin A–RXR-specific RXR-mediated signaling” by vitamin A5 is an essential, nutritionally relevant, micronutrient-mediated, and physiologically necessary effect of vitamin A; thus, it meets all the required criteria established by the WHO [5,6,79]. Consequently, vitamin A5 can be clearly classified as a vitamin and also as a new vitamin with specific effects not only mediated by vitamin A1 pathways.

As vitamin A5 is structurally similar to vitamin A1, it should logically be assigned as a vitamin A1-independent subclass of the general vitamin A family.

The problem is that the term “vitamin A1” is, and has often been, mistakenly equated with the term “vitamin A”, and a subclassification of the concept of vitamin A5 should only apply to the correct term “vitamin A” and not to the term “vitamin A1”, which is also mistakenly equated with vitamin A. This can lead to confusion with the current definition of vitamin A. Therefore, the definition of vitamin A should first be optimized, i.e., made more specific. This could be achieved by national and international professional bodies and authorities, such as the EFSA and the WHO.

## 10. The Occurrence of Vitamin A5 in the Human Organism

Vitamin A5 alcohol, i.e., 9CDHROL, has only been detected at very low concentrations in human serum, at 0.9 ng/mL (for comparison, all-*trans*-retinol is present at concentrations around 500–1000 ng/mL [18]). It is planned to investigate 9CDHROL and 9CDHROL esters in human sera and organs in the near future, using a newly established HPLC method. Moreover, 9CDHRA has already been detected in humans in three studies, with concentrations in human serum of 4.8 ng/mL [60], 4.2 / 4.8 ng/mL [61], and 3.9 ng/g in human adipose tissue in the EU NUTRITECH cohorts. These data have partly been published [80] and are publicly available via the EU NUTRITECH databases [81]. For comparison, all-*trans*-retinoic acid / vitamin A1 acid is present in the human organism at concentrations of 0.8–2.8 ng/mL [18].

**Figure 4:**
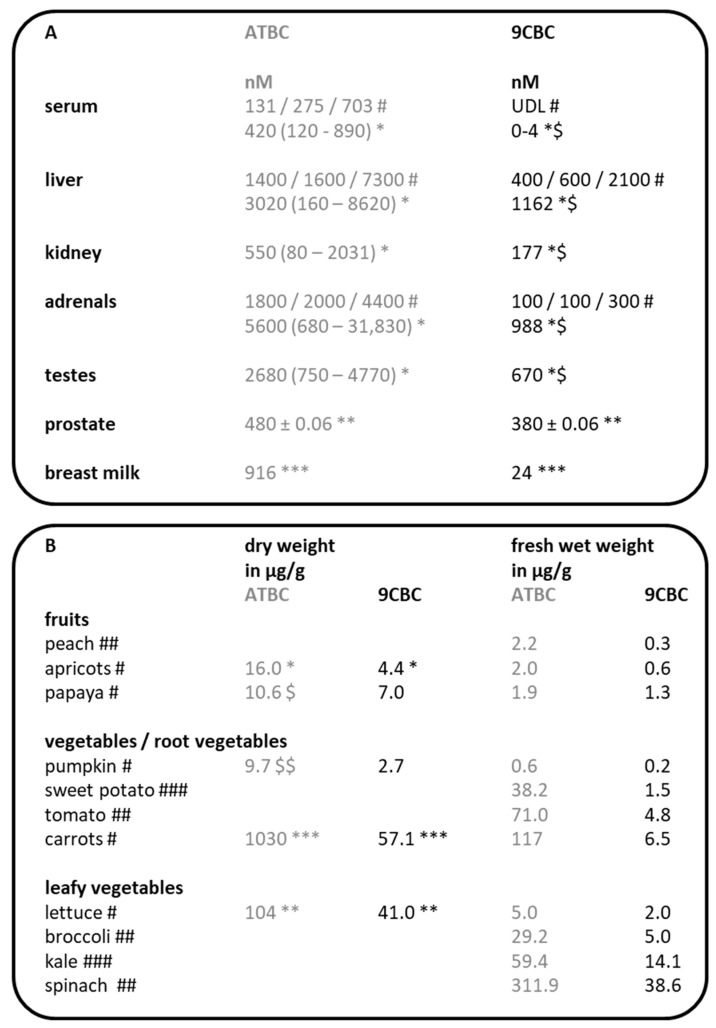
(**A**) Slightly modified from a previous reference (Table 2 in [31]), containing the concentrations of 9CBC/provitamin A5 (in nM) in different compartments in humans. Abbreviations: all-*trans*-β,β-carotene (ATBC) and 9-*cis*-β,β-carotene (9CBC). UDL—below the detection limit. The additional details added (#, $, *, **, ***) are from the table in the original article and refer to cited studies and procedures performed [18]. (**B**) Occurrence of vitamin A5 in the form of provitamin A5 in the human food chain, modified from (Table 2b in [31]). Abbreviations: all-*trans*-β,β-carotene (ATBC) and 9-*cis*-β,β-carotene (9CBC). The additional details added (*, **, ***, $, $$, #, ##, ###)) are taken from the original article and refer to cited studies and procedures performed [18].

The detection of provitamin A5 / 9CBC in the human organism has been carried out in several studies, and these data have been summarized in a review (Figure 4A; Table 2 in [31]).

## 11. Occurrence of Vitamin A5 in the Human Food Chain

In human food, 9CDHROL has only been detected at very low levels—for example, in beef liver at 8 ng/g. A further initiative and screening of fresh and processed foods will take place in the near future.

Many fresh and processed foods have already been analyzed for provitamin A5 (Figure 4B) [31]. Briefly, provitamin A5, in the form of 9CBC, is mainly found in vegetables, with a focus on leafy and root vegetables [31].

## 12. A First Proposed List of Dietary Guidelines for Vitamin A5 with Special Consideration of Provitamin A5

An assessment of actual dietary intakes and dietary guidelines for vitamin A5 alcohol and its esters cannot be carried out due to the limited data available. It is currently assumed that these forms of vitamin A5 have little or no relevance for the overall intake of vitamin A5.

**For provitamin A5, two different approaches were used to calculate possible dietary guidelines**:

**(a) Calculation of 9CBC based on its percentage contribution to the total intake of β-carotene (BC)** from natural BC-containing sources and from food sources to which BC has been added as food additives (E160a I-IV).

BC intakes are available in many databases and have been published in reviews [15]. These data can then be converted and adjusted relatively easily. This results in a calculated daily intake of 1.1 (range: 0.5–1.8) mg 9CBC / day [31], with an intake of 4.8 (range: 3–10) mg / day of total β-carotene [15]. In addition, intakes of foods with medium or high levels of 9CBC were calculated, resulting in estimated daily intakes of, for example, 1800 g of peaches or 30 g of raw spinach to achieve an intake of 1.1 mg of 9CBC / provitamin A5 per day.

**(b) A further calculation of the 9CBC intake was based on a healthy and recommended “5 A DAY” diet in humans**, and on the assumption that sufficient bioactive compounds, including micronutrients, are provided by fruits / vegetables. Cohort studies have confirmed that long-term health, longevity, and low mortality are associated with this recommended diet [82]. The 9CBC intake was calculated based on five portions of 80 g of fruits or vegetables / day, using the respective 9CBC concentrations in fruits / vegetables and taking into account the respective 9CBC concentrations in these food components and the average intake of fruits / vegetables in different European countries.

**Figure 5:**
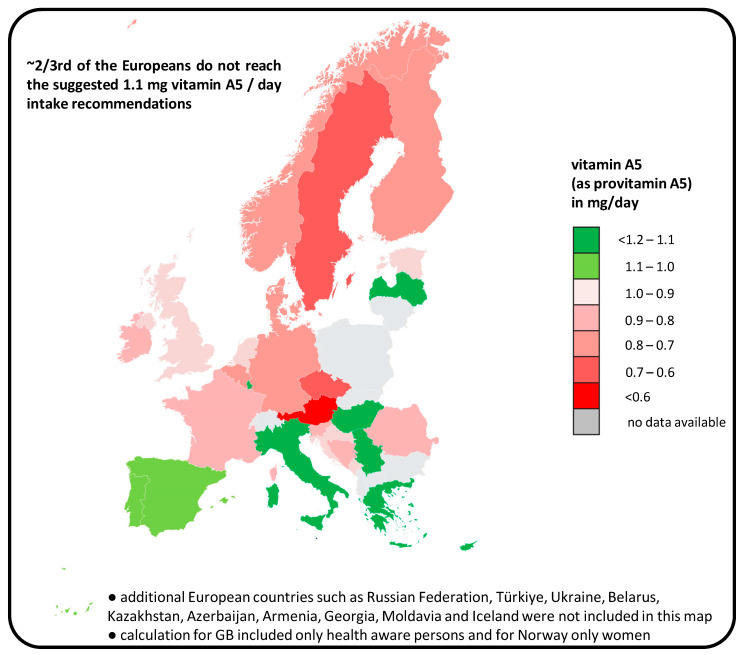
Calculated intake of vitamin A5 by Europeans [64] based on the suggested 1.1 mg vitamin A5 / day intake recommendations [31].

The calculated amount for people following the recommended “5 A DAY” diet is an average intake of 1.1 mg / day, confirming our previous calculations, so that the value of 1.1 mg 9CBC / provitamin A5 per day could be calculated consistently using two different methods of calculation [64] (Figure 5).

It should also be noted that large parts of the population, i.e., ~2 / 3 and also the population average, consume below the optimal calculated intake of 1.1 mg provitamin A5 / day [64]. This is mainly due to the low intake of fruits / vegetables, especially leafy vegetables, in many northern and central European countries [83] and the low intake of the young population. In southern and eastern European countries, where vegetable consumption is higher, the daily intake of 9CBC is sufficiently high [64].

The actually recommended intake of 1.1 mg 9CBC / provitamin A5 per day was estimated by two independent methods, while the broader general human relevance; expected *inter*-individual variability, especially of provitamin A5 intake; a broader evaluation of suggested RDIs; and the general nutri-kinetics of vitamin A5 must still be examined in detail. However, this is also partly unknown and is relevant for other vitamins [64].

Based on the low acceptance rates of a recommended “5 A DAY” diet of only 10–30% in the Western population, it was calculated, with relevance for European countries and based on the EPIC databases of 66,544 people, that ~2 / 3 of the European population are likely to have a possible vitamin A5 deficiency (Figure 5), with possible negative consequences for health, especially mental health.

## 13. What Is a Potential Vitamin A5 Deficiency? Is Vitamin A5 Essential?

Vitamin A5 deficiency is part of a general vitamin A deficiency, with congruence to a vitamin A1 deficiency based on reduced / prevented RAR—RXR-mediated signaling. It should be noted that the RXR functions as the master switch to allow multiple signaling events with other nuclear hormone receptors (NHRs), such as the vitamin D receptor (VDR), the lipid mediator receptor (peroxisome proliferator-activated receptor/PPAR), the cholesterol derivative receptor (liver X receptor / LXR), the thyroid hormone receptor (TR), the retinoic acid receptor (RAR), and NR4A2. All these processes are therefore dependent on the endogenous RXR ligand and therefore on vitamin A5, which must be obtained from the diet.

The vitamin A-mediated effect on visual perception in vitamin A5 deficiency, mediated by a non-nuclear hormone receptor (NHR)-mediated pathway, was hereby excluded, and we focused only on the effects mediated by these NHR-dependent pathways.

**Figure 6:**
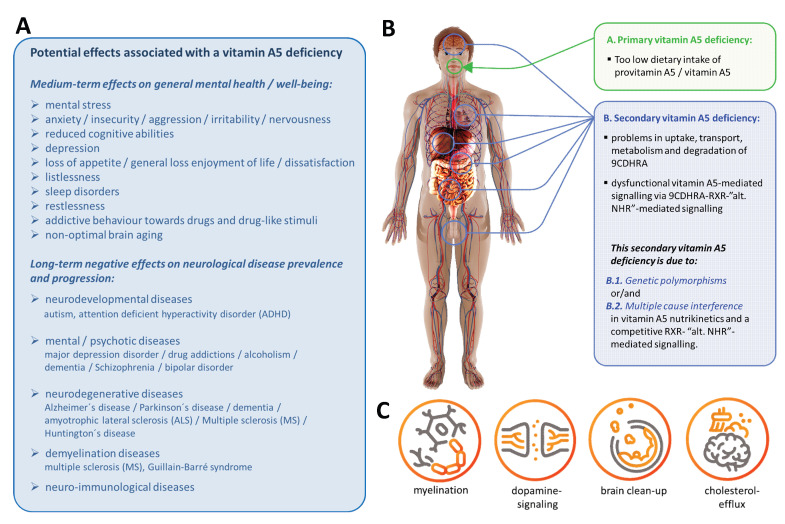
(**A**) Summary of the possible effects of a vitamin A5 deficiency [23], (**B**) Mechanisms of a primary and secondary vitamin A5 deficiency [23], (**C**) Relevant physiological mechanisms for vitamin A5-mediated effects in the brain and nerves.

A common vitamin A5 deficiency corresponds to two possible types of vitamin deficiency: **First**, a dysfunction of vitamin A1—RAR—RXR-mediated effects, therefore being fully consistent with vitamin A1 deficiency, and **second**, a dysfunction of vitamin A5—RXR—alternative NHR-mediated signaling, corresponding to a specific vitamin A5-dependent deficiency (Figure 6).

RXR is a NHR that is required as a heterodimer binding partner to interact with a variety of other nuclear receptors and to control genetic transcription and the synthesis of specific proteins. This RXR requires activation by a ligand. This RXR as a partner receptor must interact with another receptor and then bind to the DNA, and the activation of the RXR is absolutely necessary [84] in order to initiate RXR action mediation. To date, there is no plausible alternative to this activation by the endogenous vitamin A5 acid (9CDHRA) and its dietary precursor, vitamin A5, to enable this RXR-specific signaling in the human organism. As a consequence, it seems clear that vitamin A5 is thereby essential.

Recently, a logical compilation of vitamin A5—RXR-mediated signaling in relation to human health, with a focus on mental health, has been carried out [23]. In that publication, several non-optimal mental states and neurological diseases that correspond to a possible vitamin A5 deficiency were listed (Figure 6A). All of these mental states and neurological diseases involve a problem in RXR-mediated signaling [23,34,35,36] and are based on a primary (low dietary vitamin A5 intake) or secondary (impaired RXR—vitamin A5 signaling) vitamin A5 deficiency (Figure 6B). The listed physiological mechanisms (Figure 6C) have been defined here as key mechanisms of a specific vitamin A5—RXR signaling with relevance for nerves and brain, summarized in Banati et al. [23]. A specific vitamin A5 deficiency is not of relevance for RXR–RAR-mediated effects, but for “LXR / PPAR / VDR / NR4A2”—RXR-mediated effects [23].

A potential long-term undersupply towards a manifested vitamin A5-deficiency is a difficult task to determine. As for other vitamins and micronutrients, based on national and international governmental organizations guidelines, for a daily intake were suggested for specific subgroups like children, elderly and pregnant women as well as simplified and broader recommendations for the whole population [85]. In these broader calculations, adjustments for body weight and the altered, typically faster metabolism of children, as well as considerations of habitual intakes in these populations, have been embedded to ensure a broader relevance for the entire national or international population [86]. A personal dietary intake under these set recommendations does not in consequence mean that an immediate vitamin-specific deficiency will occur. Defined and common relevant parameters and guidelines after which long- or short-term undersupply and after which a percentile threshold of undersupply a specific vitamin deficiency, and in our case a vitamin A5 deficiency, will manifest in the human body are hardly generally to predict and depend on various individual parameters.

**Evidence that vitamin A5 mediates important functions in the brain and nerves and that vitamin A5 is an essential micronutrient are based on the following:**

**(a) Animal experiments in which this RXR signaling can specifically be activated by vitamin A5**, but not by vitamin A1.

**(b) Animal models in which the RXR has been rendered non-functional** or in which heterodimer binding partners of the RXR, such as the VDR, the cholesterol derivative receptor (LXR), the lipid mediator receptor (PPAR), and NR4A2 have been rendered non-functional, as well as where severe damage to functions in the brain and nervous system has been demonstrated. This corresponds to the mechanisms of the following diseases (Figure 6A). This allows us to further conclude that vitamin A5, while the only plausible dietary precursor of the endogenous RXR ligand, is an essential micronutrient.

**(c) The ligand activation of the RXR has been determined to be absolutely necessary for transmitting RXR-mediated signaling** in the organism, concluding thereby that vitamin A5 is essential [84].

**(d) Polymorphisms of proteins that are important for RXR signaling** and are therefore risk factors for direct and indirect physiological effects on the mechanisms, leading to specific diseases (Figure 6A).

**(e) A direct association between a low intake of foods high in 9CBC / provitamin A5 and a high prevalence of the diseases and indicators of suboptimal mental health** [25].

There seems to be clear evidence of a direct influence of vitamin A5 on important functions in the brain and nerves [23] and that vitamin A5 is thereby essential. However, a direct link between vitamin A5 and a healthy brain and physiologically important, specific brain functions in humans still needs to be experimentally confirmed in complex clinical trials in humans.

Direct evidence for the influence of vitamin A5 / provitamin A5 on RXR signaling in general is currently available in humans (summarized in [23,31]), as listed below. However, direct specific evidence for the important functions of vitamin A5 on the brain and nervous system in humans is still lacking.

## 14. Clinical Trials with Provitamin A5 and Synthetic RXR Ligands: Vitamin A5, RXR and Human Relevance

**(i) Interventions with provitamin A5-enriched extracts**: Several supplementation trials with provitamin A5- / 9CBC-enriched extracts have been carried out (summarized in [31]). Noteworthy is a study of provitamin A5 supplementation described by Shaish et al. [87], who showed a significant increase in the direct RXR-LXR-dependent factor, HDL cholesterol, after provitamin A5 supplementation. This shows that provitamin A5 supplementation can induce an RXR-mediated change in the human organism. This confirms the human nutritional relevance of the vitamin A5—RXR signaling concept through provitamin A5 / vitamin A5 supplementation.

**(ii) Synthetic RXR ligand interventions**: To date, a large number of interventions with synthetic RXR ligands (LGD-1069 / Targretin / Bexarotene) have been used in multiple sclerosis [88,89], and RXR ligands are regarded as a promising strategy for other neurological diseases such as Alzheimer’s disease [90,91,92,93,94], with broad clinical potential. Unfortunately, there is also a moderate side effect, hypertriglyceridemia, induced by this synthetic RXR ligand [88]. These side effects therefore limit the clinical use of synthetic RXR ligands in the treatment of a variety of neurological diseases.

However, these effects can easily be explained by RAR—RXR-mediated effects, which represent feedback with negative consequences for the human organism in response to high doses of an RXR ligand [95,96]. Clinical interventions with provitamin A5, and, as expected, also with vitamin A5 directly, have not caused any of these toxic effects due to their prodrug mode of action.

## 15. Vitamin A5 and the Overall Concept of Nutrition and Health

Many pieces of the puzzle are still missing to explain the health benefits of a balanced and healthy diet rich in fruit and vegetables. Today, such a balanced diet appears to be essential to provide the human body with all crucial micronutrients, including those that may still be unknown. The potential shortcomings of macro- and especially micro-nutrients in the Western diet, co-occurring with an oversupply of various macro-nutrients including carbohydrates and fats, are the hallmarks of an unbalanced Western diet [97].

Many diseases associated with the Western lifestyle, such as disorders of lipid, glucose, and insulin homeostasis and of adipocyte metabolism, with implications for **diabesity** (the term diabesity describes the global epidemic characterized by the simultaneous occurrence of obesity and type 2 diabetes) and **arteriosclerosis**; differentiation, proliferation, and apoptosis with relevance to cancer; and the regulation of the immune response with relevance to **allergies, viral, and bacterial infections**, the mechanisms already listed in Figure 6C with relevance to a variety of **neurological diseases / mental disorders** [23] are subject to this control of vitamin A5—RXR-mediated and -controlled signaling and are thus, partly or even fully, caused by a suboptimal vitamin A5-rich diet.

Vitamin A5 is a new vitamin and the precursor of the endogenous human-relevant ligand for the RXR—the “master switch” that enables this multitude of NHR-mediated effects to be activated and modulated by targeted dietary intake.

Dietary recommendations that focus exclusively on a healthy and varied diet are well-intentioned, but unfortunately, the willingness of the entire population to follow these recommendations is lacking [64,98]. Furthermore, it should not be forgotten that in Western societies, food fortification and food supplements with a variety of specific micronutrients are also important components of the diet to ensure an adequate supply of these specific micronutrients for the entire population [99,100]. Iodine may be the most prominent example [101] and, of course, vitamin A1 mainly in the form of provitamin A1 [31] is also of high importance and is added as a food ingredient, resulting in a high percentile dependence for total daily vitamin A intake [100].

The use of additional dietary supplements has not yet been shown to provide any overall general additional health benefits [102]. This may be because one or more pieces of the puzzle are still missing to replace and specifically induce these desired effects of a varied and healthy diet. A major problem is that simply adding “missing” and “crucial” micronutrients as food fortification and dietary supplements to a Western diet with strong oversupply of specific carbohydrates and fats and with additional non-optimal lifestyle parameters is just partly targeting the problem of a non-healthy and unbalanced diet.

The identification of vitamin A5 as an important, if not the most important, component of fruit and vegetables is a crucial step in explaining and communicating the health-promoting and health-maintaining effects of a healthy and varied diet. The overall aim for a better optimal healthy diet associated with lower incidences of a long list of associated diseases is based on various adaptations and not just one targeted optimization. Vitamin A5 might be a “key” component to target selective undesired health consequences of brain and nerves resulting from a long-term unbalanced Western diet. We suggest and summarize a scientific rationale for a targeted solution to prevent these specific health-related consequences resulting from the Western lifestyle and mainly from a commonly ingested unbalanced Western diet.

## 16. Summary of the Status of Vitamin A5 and Association as a New Vitamin

1. According to the WHO / FAO definition of micronutrients, and vitamins in particular, vitamin A5 is a vitamin, part of the vitamin A family, and an independent subcategory of vitamin A, with its own specific non-vitamin A / A1-mediated mode of action, which corresponds to an important, if not the most important, subset of vitamin A-mediated actions: the mediation of vitamin A-specific RXR-mediated signaling.

2. Aspects still lacking are valid reference values for vitamin A5 derivatives in the human organism and recommended intake levels of vitamin A5. These values are in fact already available in sufficient quantities for provitamin A5 and could be disseminated by professional societies in suggested reference amounts and intake amounts for vitamin A5.

3. Clinical relevance in humans is partly lacking. In such studies, a specific marker of vitamin A5 deficiency / risk / undersupply (e.g., a functional transcriptomic marker or myelination) should lead to a reversible improvement with vitamin A5 supplementation. However, such biomarkers are currently lacking, as is the case for many other vitamins, such as vitamin A1 and vitamin D.

Points (2) and (3) listed are independent of point (1) to declare a vitamin. However, points (2) and (3) are necessary to declare a food as either (a) common food, (b) a food ingredient / food enriched with a food ingredient, or (c) a food supplement in a legally sound and binding manner with specific dietary reference values for vitamin A5 and vitamin A5-specific “health claims”.

## 17. Future Perspectives of Vitamin A and Vitamin A5

There is currently a pressing need to adapt to the official definition of vitamin A and further optimize the situation by using the correct vitamin A terminology. Thus, we can direct action at both national and international levels through professional societies and regulatory authorities (e.g., the EFSA and even the WHO / FAO), as well as EU legislators, whose draft laws are even implemented with binding legal relevance for food labeling, food fortification, and dietary supplements across EU member states and associated third countries.

The core issue lies in the widespread false equivalence of the term “vitamin A” with the terms “vitamin A1” and “retinol”. Over the decades, this conflation has become entrenched within numerous national and international organizations and now serves as the basis for legally binding food labeling regulatory and safety regulations in both national and international legislation.

A scientifically accurate representation of facts—in line with definitions provided by the WHO / FAO and EFSA—has direct implications not only for academic research, dietary recommendations, and science communication but also for evidence-based dietary guidelines and the commercial implementation of present and emerging scientific findings in food products with respect to food safety and correct food / nutrient labeling. European consumer protection organizations are currently pushing to exclusively adapt to the communication of scientifically incorrect and paradoxical information to be presented on food labels, which stems from outdated and misleading definitions embedded in the EU food law and EU food declaration of the European Commission [103,104].

**Firstly**, there should be an international revision of the used simplification terms for “retinol” as already clearly outlined by the WHO / FAO and IUPAC. This will allow vitamin A5 to be correctly incorporated into the definition of vitamin A, to which it belongs.

Establishing a new vitamin concept is a long and difficult process given the legal means available today. The majority needs to be convinced of such a concept by a small, manageable group of scientists. One could add that science in general is not a democracy where the majority decides, but that the majority must accept the correct scientific principles.

**Secondly**, more data are needed to prove and establish a deficiency situation in humans or to describe it accurately in animal models. Further studies on the occurrence of vitamin A5 in multiple forms and active metabolites in human food and in the human organism should also be expanded experimentally. Correlations between vitamin A5 concentrations and biomarkers of general health may be analyzed in existing cohort studies. Furthermore, associations between intake amounts and disease incidence may provide indirect evidence for the new vitamin A5 concept.

## 18. Summary for a Recommended Healthy and Varied Diet Relevant for Vitamin A5 (Figure 7)

**(1) A sufficient daily intake of vitamin A5 is advised, and is especially advised to obtain it from a varied and healthy diet. Due to the advice of national and international food and health organizations not being well-adopted by large parts of the public, the option of using food additives and dietary supplements to prevent vitamin A5 undersupply, insufficiency, and a potential vitamin A5 deficiency must also be considered**.

**Figure 7:**
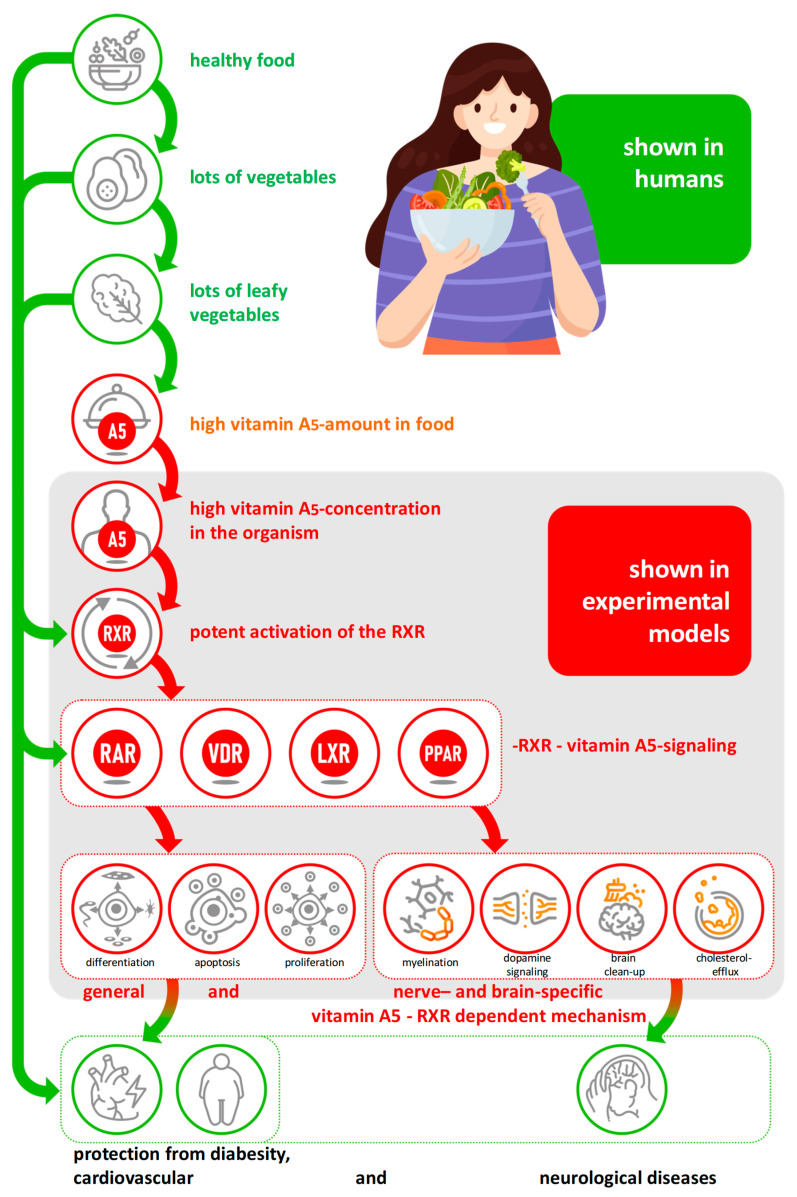
Summary of the effects of vitamin A5: from intake to nutri-kinetics, the mechanism of action to preventive effects on health based on a “step by step” confirmed cascade.

This strategy of additional supplementation with specific micronutrients in the case of an unbalanced diet with deficiencies in these specific micronutrients has recently been clearly communicated and recommended by national nutritional organizations [105] and is therefore also relevant for vitamin A5.

(2) Vitamin A5 is the dietary precursor for the activation of RXR—the optional “major switch” for multiple NHR signaling—thus, it enables and controls a variety of physiologically necessary mechanisms.

(3) Vitamin A5 is a newly identified micronutrient that plays an important role in the prevention of diet-related diseases such as diabesity, arteriosclerosis, allergies, viral and bacterial infections, neurological diseases, and mental disorders relevant to the Western lifestyle.

(4) Vitamin A5 is an important micronutrient that provides a plausible, mechanistic explanation as to why a Western lifestyle diet low in vegetables and especially leafy vegetables leads to a high prevalence of these lifestyle diseases, particularly neurological diseases and poor mental health, which can currently only be explained in a fragmentary, mechanistic way despite being observable.

## 19. General Conclusion and Limitations

This is the first proposal of a new vitamin for decades, within guidelines set up by organizations such as the WHO. More specifically, we propose this within partly diverse and misleading existing definitions of the general term vitamin A and non-existing guidelines that are needed for acknowledging a vitamin. In this review, we summarized the current status of vitamin A and vitamin A5 based on an already large range of data, adding to the existing body of evidence decades after the categorization of many other vitamins.

Due to more recent ethical restrictions, we cannot perform direct crucial data analysis; therefore, indirect alternative approaches must be considered for the categorization of vitamin A5 as a vitamin.

Further additional studies, especially for human relevance, must be added to conclusively confirm the general human relevance, including aspects covering vitamin A5 intake; different needs for vitamin A5 of various population groups; *inter*-individual variability, especially provitamin A5 intake and conversion; a broader evaluation of suggested RDIs and the general nutri-kinetics of vitamin A5 for the vitamin A5—RXR-mediated prevention of neurological and alternative diseases; and the general maintenance of good health, especially mental health.

These conclusions and potential limitations are under current discussion and evaluation of the WHO / Codex Alimentarius, the Directorate-General for Health and Food Safety of the European Commission, and the U.S. Food and Drug Administration (FDA), partly with and partly without our active participation and consultation.

We expect that a global optimization and update of the definitions of vitamins in general and vitamin A specifically, as well as the further embedding of vitamin A5 as an independent new vitamin category, will be required. In this review, we humbly provide a suggested logical strategy for global optimization not just for vitamin A, but also for other already accepted vitamin concepts.

## Data Availability

No new data were created.
